# Performance analysis of hospitals before and during the COVID-19 in Iran: A cross-sectional study

**DOI:** 10.1371/journal.pone.0286943

**Published:** 2023-06-22

**Authors:** Habib Jalilian, Seyed Mohammad Riahi, Somayeh Heydari, Masoomeh Taji

**Affiliations:** 1 Department of Health Services Management, School of Health, Ahvaz Jundishapur University of Medical Sciences, Ahvaz, Iran; 2 Department of Epidemiology and Biostatistics, Cardiovascular Diseases Research Center, School of Medicine, Birjand University of Medical Sciences, Birjand, Iran; 3 Department of Health Services Management, School of Public Health, Ahvaz Jundishapur University of Medical Sciences, Ahvaz, Iran; 4 Deputy of Management Development and Resources, Birjand University of Medical Sciences, Birjand, Iran; University of Cagliari: Universita degli Studi Di Cagliari, ITALY

## Abstract

**Background and objective:**

The COVID-19 pandemic placed considerable pressure on the health care systems and caused many disruptions to the care hospital system around the globe. This study aimed to analyze the performance of hospitals affiliated with the University of Medical Sciences and Health Services of South Khorasan Province before and during COVID-19.

**Method:**

This cross-sectional study tracked the financial performance (FP) and service performance (SP) of 12 hospitals affiliated with South Khorasan University of Medical Sciences and Health Services using the Farabar system and Hospital Information System (HIS). Our study covered two time periods: from February 2018 to February 2020 (pre-COVID-19) and from February 2020 to February 2021 (during COVID-19). SP analysis of hospitals was performed by examining the trend of monthly changes before and during the COVID-19 pandemic and analyzed using SPSS software version 22 and Paired Sample T-Test. FP of hospitals was analyzed through relevant ratios and analyzed using Microsoft Office Excel.

**Results:**

Most SP indicators decreased considerably in all hospitals during COVID-19. FP ratios (e.g., activity and leverage ratios) increased during either or both pre-COVID or COVID periods. Compared to before COVID-19, the operating margin ratio and operating expenses coverage from operating income increased from -0.50 and 66.55 to -1.42 and 41.32, respectively, during COVID-19. Moreover, the net profit margin ratio and Return On Assets (ROA) ratio were increased during COVID-19.

**Conclusion:**

COVID-19 has decreased the FP and SP of hospitals due to limitations in providing services to patients since the beginning of COVID-19. Measures such as providing various financing resources and improving the financial resilience of hospitals are essential. Funds should be disbursed to offset hospitals’ losses due to reduced elective and outpatient revenue. Policymakers should come up with holistic policies to tackle the adverse impact of such crises in the future, support hospitals financially, and consider allocating additional funding to them during emergencies.

## Introduction

COVID-19, as the most impactful contagious disease of the 21st century, has harmed public health in many countries and led to an extensive transformation of healthcare systems worldwide. Globally, the disease has led to an extensive transformation of healthcare systems and caused extensive economic damage and losses [1, 2].

The COVID-19 pandemic has posed several challenges for the health system [3, 4]. Many parts of the health system in different nations have been involved in preventing, managing, controlling, and reducing COVID-19 mortality [5]. Hospitals did not have enough time to respond appropriately to sudden changes, resulting in an unprecedented disruption to the healthcare systems [6, 7]. However, they, as a resource hub for fighting against Covid-19 in the community, have played a crucial role in providing services to patients, since the beginning of COVID-19. Due to the high disease prevalence rate and high numbers of COVID-19-related infected individuals, hospitals were provided full-capacity services, especially to COVID-19 patients [8]. Hospital managers closed outpatient wards and suspended some hospital activities [9]. Non-critical medical services, elective surgeries [10], hospital admissions, were postponed, and healthcare providers have delayed appointments [11]. Thus, the hospitals’ financial system and sustainability were seriously threatened, and the hospitals encountered a shortage of revenues and financial resources [12]. The incomes of hospitals and health systems have declined sharply due to COVID-19. This reduction has led to a sharp rise in hospital costs since the pandemic’s beginning [13]. A previous study in Iran reported, due to the COVID-19 the revenues of public hospitals decreased significantly [9].

The COVID-19 pandemic hit Iran very hard in early 2020. In mid-February 2020, Iran had the highest number of COVID-19 after China [8].

The COVID-19 outbreak in Iran coincided with the unilaterally imposed United States sanctions against the country, which has profoundly affected the country’s economy and posed many challenges to the government in terms of economics and healthcare [14]. The government injected financial aid into hospitals during the pandemic. Due to a shortage of personal protective equipment (PPE) in hospitals, the Ministry of Health and Medical Education (MOHME) purchased the equipment and made it available to hospitals [15], especially hospitals in high-prevalence provinces suffering from shortages of ICU beds. Hospitals’ FP and SP have changed the pandemic. A previous study demonstrated that hospitals’ FP (e.g. BOR and BTO) and SP decreased during COVID-19 [16].

The disease raises questions about the financial capacity of hospitals to cope with unprecedented changes in services provision [17]. Providing accurate information on the performance of hospitals before and during the COVID-19 pandemic can help policymakers evaluate and optimally allocate limited health resources to support hospitals during the pandemic. This study aimed to analyze the FP and SP of hospitals affiliated with the University of Medical Sciences and Health Services of South Khorasan Province before and during COVID-19.

## Methods

### Study design and setting

This cross-sectional study was conducted at 12 hospitals affiliated with South Khorasan University of Medical Sciences and Health Services. To estimate the impact of COVID-19 on hospitals FP and SP, we set the sample period from 20 February 2018 to 20 February 2019 and from 20 February 2019 to 20 February 2020 (pre-COVID-19), and from 20 February 2020 to 20 February 2021 (during COVID-19).

### Data collection tools

Data collection and analysis were performed in two areas; SP and FP. 1) Data related to FP, including profitability ratios, liquidity ratios, leverage ratios (solvency), activity ratios, expense ratios, and income ratios, were extracted from the software of the modern financial system and HIS of hospitals.

The organization’s FP can be assessed by looking at financial ratios obtained from financial reports published by the company. Composing financial ratios is one way to obtain useful information from financial reports [18]. There are four types of financial ratios:

1) “Profitability ratio” is the company’s ability to make a profit in relation to sales, total assets, and own capital. Profitability ratios inform how much the company’s ability to generate profits [19]. The three ratios used in profitability ratio are:
**1-a)** Return on assets = net income / assets *100
**1-b)** Net profit margin = net income / sales *100
**1-c)** Operating profit margin = operating profit / sales *100
**2)** “Liquidity Ratio” measures the company’s ability to complete its short-term obligations by looking at its current assets. The three ratios used in the liquidity ratio are:
**2-a)** “Current ratio” shows a company’s financial capacity to clear off the current obligations by using its current assets.
Formula: Current ratio = *current assets / current liabilities*
**2-b)** “Quick ratio” determines a company’s current available liquidity.
Formula: Quick ratio = *current assets—inventory—prepaid expenses / current liabilities*
**2-c)** “Cash or equivalent ratio” measures a company’s most liquid assets, such as cash and cash equivalent to the entire current liability of the concerned company.
Formula: Cash ratio = *cash & equivalent / current liabilities*
**3)** “Leverage / Solvability Ratio” measures the amount of debt a company incurs.
**3-a)** Ratio of total cost to total asset = *total cost / total asset*
**3-b)** Debt to asset ratio = total debt * 100 / total asset
**3-c)** Current debt ratio = current debt * 100 / current asset
**4)** “Activity Ratio” measures the company’s effectiveness in controlling the company’s assets [20].
**4-a)** Receivables turn over = average accounts receivable * 365 / credit sales
**4-b)** Asset turnover = *net revenue/ average total assets*
**4-c)** Inventory turnover ratio = *cost of goods sold / average inventory*
**4-d)** Average inventory = inventory at the beginning of the period + inventory at the end of the period) / 2
**4-e)** Turnover period = *360 / turnover frequency*
**4-f)** Average inventory = *inventory at the beginning of the period + inventory at the end of the period / 2*
**4-g)** Turnover period = *360 / turnover frequency*
**4-h)** Percentage of bed occupancy = occupied bed day / total bed day * 100
**4- I)** Average length of stay = *total hospitalization days / total number of inpatients*
**4-j)** Hospitalization bed day = *total days of bed occupancy during a given period*
**4-k)** Bed turnover = *number of discharges and deaths over a given period of time/number of active beds*

"Expense ratio" measures the amount and combination of different sources of cost. This ratio in our study encompassed: a) salary share of the total cost, b) share of medicine and medical supplies consumed of the total cost, c) cost ratio to active bed, d) cost ratio to inpatient bed day, and e) cost coverage from revenue = operating income * 100 / operating cost

"Income ratio" measures the amount and combination of different sources of revenue. In our study, this ratio included: a) the share of hospitalization income of total income, b) the share of outpatient income of total income, c) income per active bed, d) income per hospitalization bed day, e) outpatient income per outpatient, and f) inpatient income per inpatient.

2) Data related to SP, including BOR, BTO ratio, number of outpatients, number of inpatients, number of surgeries, deaths, Average Length of Stay (ALOS), and inpatient bed days, were extracted from the Farabar system. Data related to SP were collected from February 2018 to February 2021 monthly, and data related to FP (financial ratios) were calculated as yearly. The FP and SP of hospitals were compared before and during COVID-19.

### Statistical analysis

Data were analyzed using EXCEL version 2010 and SPSS software version 25. The normality of data was verified using Shapiro-Wilk tests. FP indicators were calculated and analyzed using EXCEL. Moreover, data related to SP were analyzed using frequency and percent, mean ± standard deviation (SD), and paired sample t-test. The trend of changes in the periods before and during COVID-19 was analyzed using SPSS 22 software.

### Ethics approval

This study was approved by the Ethics Committee of Birjand University of Medical Sciences (Reference No: IR.BUMS.REC. 1400.141). All methods were carried out in accordance with relevant guidelines and regulations.

## Results

### The results of the SP

As shown in Table 1, compared to the time before COVID-19, the number of inpatients, the number of outpatients, BOR (%), and inpatient bed days reduced significantly during COVID-19. During COVID-19, the highest percentage change was related to the number of outpatients (-40.71). The lowest percentage change pre-COVID-19 was related to BOR (-0.26), whilst during COVID-19 the percentage change of inpatient bed days was the lowest (-22.17).

**Table 1:** Comparison of the percentage of annual changes in hospitals’ SP before and during the COVID-19.

Variables	Pre- COVID-19		During the COVID-19	Pre-COVID-19 changes (%)	During COVID-19 changes (%)
	2018–19	2019–20	2020–21		
**The number of inpatients**	85230	89672	64161	5.21	-28.45
**The number of outpatients**	2049764	2006556	1189729	-2.11	-40.71
**BOR (%)**	62.38	62.22	45.38	-0.26	-27.07
**Inpatient bed day**	264.01	260.27	202.57	-1.42	-22.17

As shown in Table 2, before COVID-19, BOR and BTO were reduced in all hospitals during COVID-19. BOR reduced slightly from 62.38 to 62.22 in the years before COVID-19 and thereafter reduced again considerably to 45.38 during COVID-19. Similarly, in all hospitals, BTO saw a slight decrease in the years before COVID-19 (from 75.41 to 74.66) and a significant decrease (52.13) during COVID-19. In all studied hospitals, ALOS fluctuated during the studied periods. There was no significant association between SP indicators and the size of hospitals.

**Table 2:** Results of BOR (%), ALOS (days), and BTO of hospitals by their name and size.

Size	Hospitals’ name	BOR (%)			ALOS (days)			BTO		
		2018–19	2019–20	2020–21	2018–19	2019–20	2020–21	2018–19	2019–20	2020–21
**>200 beds**	**Emam Reza**	62.87	64.35	56.50	4.50	4.41	4.82	53.85	49.98	38.84
	**Valiasr**	80.94	74.43	49.51	3.48	3.31	3.81	86.79	81.25	49.73
**100–200 beds**	**Shohada**	57.33	64.32	49.83	2.16	2.20	2.94	92.15	102.57	68.20
	**Mostafa Khomeini**	66.93	63.88	49.69	2.38	2.38	2.41	102.69	98.14	75.61
	**Razi**	56.79	67.86	44.48	2.93	3.57	3.43	64.23	64.90	48.29
**<100 beds**	**Ali Ibn Abitaleb**	35.82	48.19	32.38	2.18	2.21	1.88	62.68	83.88	63.54
	**Khatam-al- Anbya**	22.54	42.07	14.50	1.65	1.92	1.52	47.30	79.61	34.20
	**Emam Ali**	37.79	38.06	25.58	2.21	2.01	1.77	62.47	68.89	52.73
	**Shafa**	43.64	52.50	42.19	2.43	1.98	1.93	56.22	63.16	51.40
	**Chamran**	61.78	54.35	45.75	2.59	2.51	2.53	82.71	75.75	64.23
	**Atashdoost**	54.01	49.60	41.62	2.15	1.90	1.83	90.24	90.08	76.87
	**Hazrat Rasool**	58.14	52.38	62.22	2.89	2.81	2.92	67.26	64.96	74.66
**Total SP of hospitals**		62.38	62.22	45.38	3	2.92	3.11	75.41	74.66	52.13

Tables 3 & 4 showed the Paired sample t-test results of SP indicators by hospitals’ size and name. According to the results, the mean total of inpatient bed days, BOR, and BTO reduced from 22356 days, 63.9%, and 6.4 before the COVID-19 to 16655 days, 45.2%, 3.1 and 4.3, respectively, during the COVID-19. In contrast, the AOLS increased from 2.9 days before COVID-19 to 6.8 days during COVID-19 (Table 3).

**Table 3:** Paired sample t-test results of SP indicators (monthly) by hospitals’ size and name.

Size	Hospitals	Pre- COVID-19 and during COVID-19	Inpatient bed day (D)	BOR (%)	ALOS (day)	BTO (Patients)
**>200 beds**	**Emam Reza**	**Pre- COVID-19**	3795 ± 355**	65.69 ± 4.2**	4.3 ± 0.4*	4.3 ± 0.37***
		**During COVID-19**	3142 ± 677	54.6 ± 9.6	4.8 ± 0.4	3.2 ± 0.52
	**P-value**	**Pre- COVID-19**	.00	.01	.01	.00
		**During COVID-19**				
	**Valiasr**	**Pre- COVID-19**	6065 ± 542***	76.9 ± 5***	3.3 ± 0.2*	6.9 ± 0.5***
		**During COVID-19**	4246 ± 1059	49.7 ± 9.5	3.7 ± 0.6	4.2 ± 0.7
	**P-value**	**Pre- COVID-19**	.00	.00	.03	.00
		**During COVID-19**				
**100–200 beds**	**Shohada**	**Pre- COVID-19**	2324 ± 271	65.3 ± 5.9***	2.1 ± 0.1**	8.7 ± 0.8***
		**During COVID-19**	2096 ± 427	50.6 ± 4.2	2.9 ± 0.6	5.7 ± 0.5
	**P-value**	**Pre- COVID-19**	.13	.00	.00	.00
		**During COVID-19**				
	**Mostafa Khomeini**	**Pre- COVID-19**	2430 ± 185***	66 ± 5***	2.3 ± 0.1	8.4 ± 0.7***
		**During COVID-19**	1635 ± 314	49 ± 9	2.4 ± 0.3	6.2 ± 0.5
	**P-value**	**Pre- COVID-19**	.00	.00	.87	.00
		**During COVID-19**				
	**Razi**	**Pre- COVID-19**	3041 ± 434**	69.5 ± 7***	3.5 ± 0.2	5.6 ± 0.5***
		**During COVID-19**	2404 ± 496	44.5 ± 7	3.5 ± 0.3	3.9 ± 0.8
	**P-value**	**Pre- COVID-19**	.00	.00	.64	.00
		**During COVID-19**				
**<100 beds**	**Ali Ibn Abitaleb**	**Pre- COVID-19**	383 ± 84***	49.6 ± 9.8***	2.2 ± 0.1***	7 ± 1.3***
		**During COVID-19**	239 ± 51	32.5 ± 6.2	1.9 ± 0.1	5.2 ± 1
	**P-value**	**Pre- COVID-19**	.00	.00	.00	.00
		**During COVID-19**				
	**Khatam-al- Anbya**	**Pre- COVID-19**	377 ± 55***	44.18 ± 5.9***	1.9 ± 0.1***	6.9 ± 0.7***
		**During COVID-19**	116 ± 32	14.48 ± 3.9	1.5 ± 0.1	2.8 ± 0.7
	**P-value**	**Pre- COVID-19**	.00	.00	.00	.00
		**During COVID-19**				
	**Emam Ali**	**Pre- COVID-19**	372 ± 69***	39.45 ± 6.9***	2 ± 0.1*	5.8 ± 0.8***
		**During COVID-19**	218 ± 74	25.62 ± 7.2	1.7 ± 0.3	4.3 ± 0.8
	**P-value**	**Pre- COVID-19**	.00	.00	.02	.00
		**During COVID-19**				
	**Shafa**	**Pre- COVID-19**	338 ± 81	51.92 ± 10	1.98 ± 0.3	5.2 ± 0.8
		**During COVID-19**	266 ± 146	42.62 ± 13.3	1.86 ± 0.9	4.5 ± 1.2
	**P-value**	**Pre- COVID-19**	.15	.06	.06	.09
		**During COVID-19**				
	**Chamran**	**Pre- COVID-19**	1158 ± 237***	56.3 ± 6.7***	2.6 ± 0.2	6.4 ± 0.6***
		**During COVID-19**	802 ± 180	42.8 ± 8.4	2.4 ± 0.3	5.1 ± 0.9
	**P-value**	**Pre- COVID-19**	.00	.00	.18	.00
		**During COVID-19**				
	**Atashdoost**	**Pre- COVID-19**	557 ± 59***	50.72 ± 5.2***	1.9 ± 0.2	7.6 ± 0.8***
		**During COVID-19**	437 ± 74	41.57 ± 5.8	1.8 ± 0.2	6.3 ± 0.5
	**P-value**	**Pre- COVID-19**	.00	.00	.31	.00
		**During COVID-19**				
	**Hazrat Rasool**	**Pre- COVID-19**	986 ± 109***	54.25 ± 4.6***	2.8 ± 0.1*	5.6 ± 0.6***
		**During COVID-19**	624 ± 184	30.9 ± 8.7	3.5 ± 0.8	2.6 ± 0.5
	**P-value**	**Pre- COVID-19**	.00	.00	.01	.00
		**During COVID-19**				
**Total**		**Pre- COVID-19**	22356 ± 1471***	63.9 ± 2.6***	2.9 ± 0.08*	6.4 ± 0.3***
		**During COVID-19**	16655 ± 2610	45.2 ± 4.8	3.1 ± 0.3	4.3 ± 0.5
**P-value**		**Pre- COVID-19**	.00	.00	.02	.00
		**During COVID-19**				

* P < 0.05 was considered as significant

** P < 0.01 was considered as significant

*** P < 0.001 was considered as significant

**Table 4:** Paired sample t-test results of SP indicators (monthly) by size and hospitals’ name.

Size	Hospitals	Pre- COVID-19 and during COVID-19	Outpatients (N)	Inpatients (N)	Surgeries (N)	Deaths (N)
**>200 beds**	**Emam Reza**	**Pre- COVID-19**	31896 ± 2634***	879 ± 86***	579 ± 69**	10.8 ± 5.2
		**During COVID-19**	20440 ± 3686	649 ± 124	460 ± 112	9.8 ± 4
	**P-value**	**Pre- COVID-19**	.00	.00	.00	.60
		**During COVID-19**				
	**Valiasr**	**Pre- COVID-19**	25667 ± 2461***	1825 ± 126***	447 ± 50***	36 ± 4
		**During COVID-19**	8362 ± 2072	1124 ± 221	242 ± 68	47 ± 25.9
	**P-value**	**Pre- COVID-19**	.00	.00	.00	.14
		**During COVID-19**				
**100–200 beds**	**Shohada**	**Pre- COVID-19**	25601 ± 1828***	1100 ± 119***	420 ± 82	13 ± 4
		**During COVID-19**	11580 ± 2357	755 ± 81	352 ± 85	12 ± 7
	**P-value**	**Pre- COVID-19**	.00	.00	.88	.68
		**During COVID-19**				
	**Mostafa Khomeini**	**Pre- COVID-19**	13746 ± 739***	1056 ± 92***	428 ± 67*	14 ± 2.3
		**During COVID-19**	7392 ± 2270	726 ± 108	365 ± 65	17 ± 11.5
	**P-value**	**Pre- COVID-19**	.00	.00	.03	.33
		**During COVID-19**				
	**Razi**	**Pre- COVID-19**	4702 ± 967	780 ± 208	413 ± 59	15.6 ± 5
		**During COVID-19**	5566 ± 1351	654 ± 165	369 ± 120	19 ± 5
	**P-value**	**Pre- COVID-19**	.40	.07	.27	.10
		**During COVID-19**				
**<100 beds**	**Ali Ibn Abitaleb**	**Pre- COVID-19**	7298 ± 754***	173 ± 32***	2.2 ± 1.6***	0.7 ± 1
		**During COVID-19**	4599 ± 718	126 ± 20	14.7 ± 6.2	0.15 ± 0.5
	**P-value**	**Pre- COVID-19**	.00	.00	.00	.14
		**During COVID-19**				
	**Khatam-al- Anbya**	**Pre- COVID-19**	4352 ± 438***	194 ± 20***	40.5 ± 24***	0.08 ± 0.3
		**During COVID-19**	2935 ± 366	77 ± 18	29.8 ± 8.6	0.08 ± 0.3
	**P-value**	**Pre- COVID-19**	.00	.00	.00	1
		**During COVID-19**				
	**Emam Ali**	**Pre- COVID-19**	10401 ± 1005***	181 ± 24***	29 ± 7**	0.25 ± 0.4
		**During COVID-19**	6636 ± 1172	120 ± 25	19 ± 8	0.58 ± 1.1
	**P-value**	**Pre- COVID-19**	.00	.00	.00	.36
		**During COVID-19**				
	**Shafa**	**Pre- COVID-19**	11381 ± 1326***	170 ± 28***	59 ± 12	1.1 ± 0.8
		**During COVID-19**	6832 ± 1088	140 ± 26	66 ± 19	1.8 ± 2.3
	**P-value**	**Pre- COVID-19**	.00	.00	.25	.36
		**During COVID-19**				
	**Chamran**	**Pre- COVID-19**	12516 ± 1269***	451 ± 92***	216 ± 18	2.25 ± 1.8**
		**During COVID-19**	7861 ± 1806	331 ± 59	228 ± 52	4.6 ± 1.4
	**P-value**	**Pre- COVID-19**	.00	.00	.46	.00
		**During COVID-19**				
	**Atashdoost**	**Pre- COVID-19**	11690 ± 1549***	284 ± 31***	60 ± 18**	2.5 ± 2.1
		**During COVID-19**	6001 ± 1020	223 ± 16	84 ± 15	2.5 ± 2
	**P-value**	**Pre- COVID-19**	.00	.00	.00	1
		**During COVID-19**				
	**Hazrat Rasool**	**Pre- COVID-19**	2594 ± 551**	340 ± 86***	90 ± 23***	10 ± 4
		**During COVID-19**	1852 ± 597	179 ± 40	12 ± 12.9	10 ± 7
	**P-value**	**Pre- COVID-19**	.01	.00	.00	.84
		**During COVID-19**				
**Total**		**Pre- COVID-19**	171830 ± 11293***	7654 ± 666***	2786 ± 221***	107 ± 17
		**During COVID-19**	98406 ± 16688	5274 ± 463	2246 ± 394	127 ± 56
**P-value**		**Pre- COVID-19**	.00	.00	.00	.24
		**During COVID-19**				

* P < 0.05 was considered as significant

** P < 0.01 was considered as significant

*** P < 0.001 was considered as significant

The mean total number of outpatients, inpatients, and surgeries reduced from 171830, 7654, and 27.86 before COVID-19 to 98406, 5274, and 22.46, respectively, during the COVID-19 (Table 4). The highest reduction was related to the number of outpatients (42%). The reduction in the number of outpatients was highest (67%) in Vali Asr hospital (the center of admission for COVID-19 patients). Furthermore, compared to before COVID-19, the mean in-hospital mortality increased from 107 before COVID-19 to 127 during COVID-19, but there were no significant differences between the mortality counts before COVID-19 with that after COVID-19 (Table 4).

### The results of FP

Table 5 presents the percentage of annual changes in the FP of studied hospitals. Compared to before COVID-19, liquidity ratios, activity ratios, and profitability ratios decreased, while leverage and expense ratios increased during COVID-19.

**Table 5:** Comparison of the percentage of annual changes in the hospitals’ FP.

Ratios	Indicators	Pre- COVID-19		During COVID-19	Pre-COVID-19 changes (%)	During COVID-19 changes (%)
		2018–19	2019–20	2020–21		
**Liquidity ratio**	**Current ratio**	1.21	0.85	0.90	-29.36	5.17
	**Quick ratio**	1	0.67	0.68	-33.15	0.62
	**Cash ratio**	0.16	0.12	0.01	-22.70	-90.32
**Leverage ratio**	**Ratio of total costs to total assets**	0.68	0.85	1.18	25.61	39.07
	**Debt to asset ratio**	35	53	70	53.85	30.65
	**Current debt ratio**	83	117	111	41.56	-4.91
**Activity ratio**	**Receivable turnover ratio**	73	64	100	-12.04	55.44
	**Asset turnover ratio**	0.75	0.69	1.14	-8.62	66.46
	**Inventory Turnover Ratio**	19.58	15.19	22.26	-22.41	46.53
	**Average inventory**	215,899	245,826	301,163	13.86	22.51
**Profitability ratio**	**Return on assets**	0.07	-0.17	-0.04	-331.53	-73.76
	**Operating profit margin**	-0.50	-0.70	-1.42	39.60	102.41
	**Net profit margin**	0.10	-0.24	-0.04	-353.36	-84.24
**Expense ratio**	**Operating expense coverage from operating income**	66.55	58.77	41.32	-11.70	-29.69
	**Operating cost per active bed (million Rials)**	2.80	3.22	4.54	15.14	40.72
	**Operating cost per inpatient day (million Rials)**	12.38	14.82	27.35	19.71	84.49
**Income ratio**	**Operating income of total revenue**	0.51	0.61	0.34	17.96	-43.76
	**Operating income per active bed (million Rials)**	1.86	1.89	1.87	1.68	-1.06
	**Operating income per inpatient day (million Rials)**	8.24	8.71	11.30	5.71	29.72

In the “*liquidity ratios*,” all three ratios were reduced during COVID-19. The highest change was related to the cash ratio, representing a change of approximately -90%. While the cash ratio experienced a slight decrease pre-COVID-19 (from 0.16 to 0.12), it decreased considerably during COVID-19. The current ratio decreased from 1.21 to 0.85 before COVID-19 and again increased to 0.9 during COVID-19. In contrast, the quick ratio did not change significantly during COVID-19 compared with before COVID-19. Although the quick ratio experienced a slight decrease before COVID-19 (from 1 to 0.67), it saw a slight growth during COVID-19 (0.68). In the “*leverage ratios*,” The highest percentage change during COVID-19 was related to the current debt ratio (-4.91%). The total costs to total assets and debt to asset ratio increased during either or both pre-COVID or COVID periods. Also, while the current debt ratio increased pre-COVID-19 (from 83 to 117), it decreased again, reaching 111 during COVID-19.

In “*activity ratios*,” the highest percentage change before COVID-19 was related to the inventory turnover ratio (-%22.41). By contrast, the highest percentage change during COVID-19 was related to the asset turnover ratio (%66.46). The receivable turnover ratio, asset turnover ratio, and inventory Turnover ratio decreased pre-COVID-19, whereas these ratios rose during COVID-19. Average inventory increased steadily during pre-COVID and COVID time periods.

In the “*profitability ratio*,” all ratios were negative during COVID-19. The operation margin ratio decreased during either or both pre-COVID or COVID periods. This ratio decreased from -0.50 in 2018–19 to -0.70 in 2019–20 before COVID-19 and dropped again more significantly during COVID-19 (reached 1.42). Both ROA and net profit margin decreased pre-COVID-19 before increasing during COVID-19 and reached -0.04. The ROA decreased from 0.07 in 2018–19 to -0.17 in 2019–20 and thereafter again increased to -0.04 during COVID-19. The net profit margin decreased from 0.10 in 2018–19 to -0.24 in 2019–20 and rose again to -0.04 during COVID-19 (Table 5).

As illustrated in Table 5, the highest percentage change in “expense ratios” was related to operating costs per inpatient day. We found that operating expenses coverage from operating income decreased during either or both pre-COVID or COVID periods. The ratio decreased from 66.55 in 2018–19 to 58.77 in 2019–20 and decreased again to 41.32 during COVID-19. By contrast, operating costs per active bed and inpatient day increased during either or both pre-COVID or COVID periods. The former saw an increase of about 1.5-fold during COVID-19 than pre-COVID time periods, and the latter saw an increase of about 2-fold during COVID-19 than pre-COVID time periods.

In “*income ratios*,” the highest percentage change pre- and during COVID-19 was related to operating income of total revenue (-43.76%%). Operating income per inpatient day experienced an increase of about 1.3-fold during COVID-19. The operating income of total revenue and operating income per active bed fluctuated during the studied periods. While the former increased from 0.51 in 2018–19 to 0.61 in 2019–20 before decreasing to 0.34 in 2020, and the latter changed marginally during either or both pre-COVID or COVID periods.

**Fig 1:**
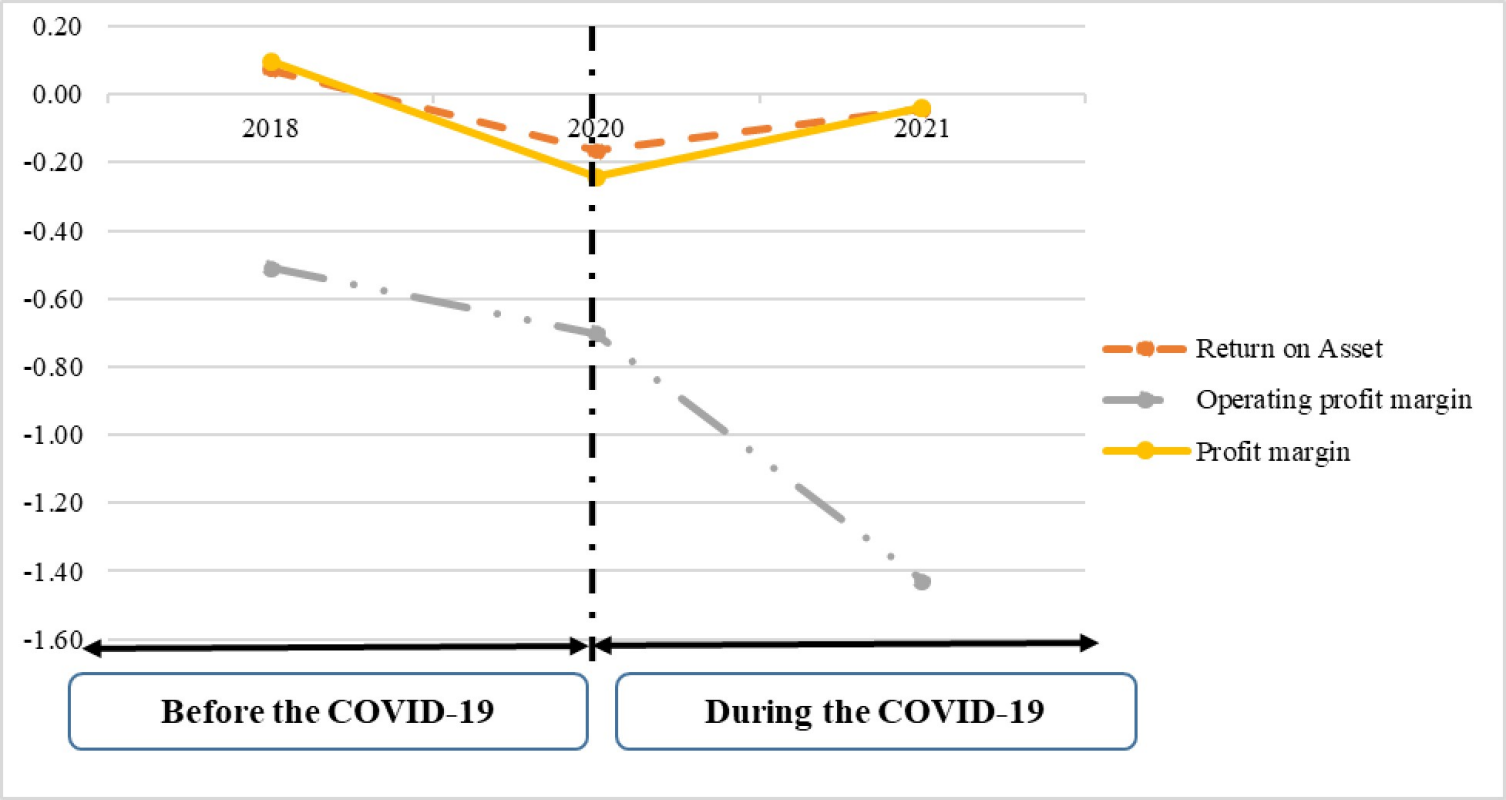
The trend of changes in FP ratios before and during COVID-19.

As illustrated in Fig 1, while the operating margin ratio decreased, net profit margin ratios and ROA in profitability ratios increased during COVID-19.

Table 6 presents the FP of each hospital by size. In this study, the total current debt ratio, cost per inpatient day, and income per inpatient day rose during either or both pre-COVID or COVID periods. The highest increase was related to the total cost per inpatient day, a 1.8-fold increase during COVID-19 compared with 2019–20 and a 2.2-fold increase compared with 2018–20. We also found that during COVID-19, total income per inpatient day increased only about 1.2-fold compared with pre-COVID-19. By contrast, the current Debt Ratio, operating profit margin, and operating cost coverage from operating income fell during either or both pre-COVID or COVID periods. The total of ROA and net profit margin fluctuated over the studied periods. Our results showed that ROA, operating profit margin, and net profit margin ratios were negative in most hospitals pre-COVID and during COVID-19. The operating profit margin in most hospitals had a downward trend, and the net profit margin fluctuated in 7 hospitals (of 12 hospitals). Also, while operating cost coverage from operating income (%) decreased steadily, income per inpatient day revealed a rising trend in most hospitals. The current ratio and current debt ratio ratios also fluctuated.

**Table 6:** FP ratios by size and hospital name.

Size	Hospitals	Year	Current ratio	Current Debt Ratio	Return on Asset	Operating profit margin	Net profit margin	Costs per inpatient day) Thousand Rials)	Operating cost coverage from operating income (%)	Income per inpatient day) Thousand Rials)
**>200 beds**	**Emam Reza**	2018–19	1.24	80.73	-0.55	-0.26	-1.26	9,393	80	7,479
		2019–20	0.88	114.19	-0.28	-0.53	-0.28	12,538	65	8,209
		2020–21	1.04	95.83	-0.20	-1.17	-0.12	21,000	46	9,691
	**Valiasr**	2018–19	1.55	64.48	0.08	-0.21	0.06	8,724	83	7,198
		2019–20	1.18	84.46	-0.34	-0.45	-0.24	11,432	69	7,903
		2020–21	0.96	104.30	-0.45	-1.28	-0.21	22,402	44	9,816
**100–200 beds**	**Shohada**	2018–19	1.19	84.00	-0.06	-0.48	-0.05	15,875	68	10,752
		2019–20	0.75	132.57	-0.47	-0.58	-0.35	15,938	63	10,097
		2020–21	0.89	112.55	-0.07	-1.31	-0.03	24,718	43	10,690
	**Mostafa Khomeini**	2018–19	1.42	70.66	0.00	-0.42	0.00	12,757	70	8,984
		2019–20	0.64	156.91	-0.24	-0.53	-0.41	14,855	65	9,679
		2020–21	0.88	113.71	0.11	-1.27	0.10	31,912	44	14,081
	**Razi**	2018–19	1.20	83.57	0.55	-0.72	0.54	19,038	58	11,050
		2019–20	0.89	112.45	-0.09	-0.52	-0.21	14,398	66	9,483
		2020–21	0.73	136.93	-0.04	-0.92	-0.06	23,994	52	12,485
**<100 beds**	**Ali Ibn Abitaleb**	2018–19	0.57	174.17	-0.07	-2.03	-0.17	36,750	33	12,110
		2019–20	0.60	165.82	-0.04	-2.08	-0.07	21,600	32	7,003
		2020–21	0.74	134.98	-0.02	-3.74	-0.02	52,708	21	11,129
	**Khatam-al- Anbya**	2018–19	0.54	185.77	-0.13	-1.50	-0.17	34,815	40	13,922
		2019–20	0.49	204.05	-0.12	-1.86	-0.11	24,246	35	8,467
		2020–21	0.75	133.86	0.11	-4.39	0.07	109,027	19	20,243
	**Emam Ali**	2018–19	0.81	123.82	-0.03	-1.05	-0.05	22,551	49	11,016
		2019–20	0.63	159.92	-0.09	-1.49	-0.15	27,492	40	11,051
		2020–21	0.81	124.11	-0.09	-2.47	-0.10	66,947	29	19,285
	**Shafa**	2018–19	1.59	62.74	0.06	-1.34	0.11	27,375	43	11,717
		2019–20	0.84	118.93	-0.13	-1.49	-0.26	35,287	40	14,150
		2020–21	0.95	105.43	0.30	-2.13	0.34	60,410	32	19,308
	**Chamran**	2018–19	0.84	118.35	-0.04	-0.90	-0.05	18,481	53	9,710
		2019–20	0.81	122.90	-0.14	-1.12	-0.21	21,889	47	10,340
		2020–21	0.91	109.49	-0.17	-1.53	-0.19	36,407	40	14,402
	**Atashdoost**	2018–19	0.95	105.12	-0.03	-0.86	-0.11	19,790	54	10,634
		2019–20	0.64	155.41	-0.01	-1.42	-0.04	28,472	41	11,774
		2020–21	0.73	136.59	0.11	-1.99	0.16	53,574	33	17,941
	**Hazrat Rasool**	2018–19	0.99	101.34	0.06	-0.84	0.22	8,782	54	4,767
		2019–20	0.92	109.17	-0.09	-1.62	-0.20	17,219	38	6,571
		2020–21	0.85	117.02	-0.17	-0.70	-0.24	14,823	59	8,704
**Total**		2018–19	1.21	82.67	0.07	-0.51	0.10	12,382	66	8,202
		2019–20	0.85	117.02	-0.17	-0.70	-0.24	14,823	59	8,704
		2020–21	0.90	111.27	-0.04	-1.43	-0.04	27,348	41	11,256

## Discussion

This study aimed to analyze the FP and SP of hospitals affiliated with the University of Medical Sciences and Health Services of South Khorasan Province before and during COVID-19. The results of hospitals’ SP and FP are discussed in turn below.

Regarding hospitals’ SP, our results showed that the number of outpatients, inpatients, and surgeries decreased significantly. The highest percentage change was related to the number of outpatients, representing a considerable decline. Previous studies demonstrated that the average annual number of outpatient visits [21–26], inpatient admission, MRI services and surgical decreased during COVID-19 [27–29]. Hung et al, reported with the rise in inpatients, the severity of COVID-19, and staffing shortages, COVID-19 has had a substantial impact on the US healthcare system [30]. Since people feared being infected with COVID-19 when visiting the hospital, they refused to go to hospitals. Even if they needed to receive health services in hospitals, they refused to visit the hospital, and the number of outpatient visits decreased considerably. At the onset of COVID-19, for better management of patients with COVID-19, most hospitals allocated their beds to these patients. Hospitals had no choice but to provide more beds for these patients, which led to fewer beds available for non-COVID-19 patients and decreased hospital revenues [31, 32]. Khullar et al showed that the reduction in outpatient visits, due to fears of COVID-19 has led to a reduction in hospital service capacity in the US [17].

One of the reasons for the decline in hospitals’ revenue in Iran was found to be decreased admissions and elective surgeries, and one of the significant reasons for this change in hospitals’ revenue was MOHME’s decision to turn hospitals into special centers for the admission and treatment of COVID-19 patients [33]. Moreover, due to a lack of manpower and their high workload during the pandemic, some hospitals managers reduced the admission of non-COVID-19 patients [34].

In our study, the hospitals’ mean inpatient bed day (D), BOR (%), and BTO (Patients) decreased significantly. Our results are in keeping with previous studies conducted in different settings [16, 35–38] which showed that BTO and BOR decreased during the pandemic. An American study demonstrated inpatient mortality from COVID-19 was associated with increased BOR [38].

In our study, the BTO and BOR figures were below the standard limit. A BTO ratio of > 5.6 means the use of a bed is more than the standard limit for one year (the standard value of the bed occupancy rate is between 60% - 85% [16]). BOR was found to be close to the minimum standard. Higher BOR is associated with higher operating profit and total profit. The Economy of Scale means more BOR leads to the distribution of fixed costs to a larger number of patients [39].

In this study, although the mortality rate in most hospitals increased during the pandemic, there was no significant difference between mortality counts before COVID-19 and during COVID-19. In 2020 mortality counts worldwide were estimated to be less than 600000 due to COVID-19, as people prevented injuries and respiratory diseases by staying at home [40]. In our study, ALOS rose during COVID-19, but this difference was not statistically significant. Our results are in line with previous studies conducted in Iran, the US, and Indonesia [16, 41, 42]. It has shown that increased inpatient hospitalization due to COVID-19 in the US contributed to a nationwide reduction in inpatient bed availability, which previous studies have reported to have the strongest correlation with Emergency Department Length of Stay for admitted (ED LoS-A); the less inpatient bed availability, the longer LoS-A [41, 43, 44]. Lucero et al demonstrated a proportional increase in lower Emergency Severity Index encounters may have contributed to an increase in LOS [45].

Regarding hospitals’ FP, all leverage and activity ratios increased during either or both pre-COVID or COVID periods. Also, operating cost per active bed (million Rials), operating cost per inpatient day (million Rials), and operating income per inpatient day (million Rials) increased. by contrast, operating expense coverage from operating income, operating income of total revenue decrease during either or both pre-COVID or COVID periods.

In the present study, operating income per inpatient day and operating cost per inpatient day rose in most hospitals. Part of this growth is because of a reduction in the number of inpatients, outpatients, and surgeries and the change in the type of services provided, in addition to the increase of service tariffs in income and inflation in costs. To contain and prevent the spread of COVID-19 and build additional capacity for hospitals and staff, many hospitals closed outpatient wards and postponed or canceled elective visits. Although these changes were needed to respond to COVID-19, they potentially threatened the financial capacity of hospitals, especially those that were already financially challenged and highly dependent on outpatient and elective revenues [17]. A decline in outpatient revenues may not be partially recouped by the higher occupancy of the hospital intensive care unit (ICU) during COVID-19 and the increase in service delivery after the end of the pandemic. An Iranian study demonstrated a reduction in the Masih Daneshvari hospital’s revenue (the center of admission for COVID-19 patients in Tehran, the capital of Iran) during the pandemic [34]. One reason for this decline can be related to the cancellation of elective surgeries and a reduction in hospital visits during COVID-19 [46].

Due to the growth of operating costs, the growth of operating incomes, and the significant mismatch between revenue growth and expenses, the university faced an increase in operating losses of 1660027 million rials (104.35%). Also, net loss decreased by 71.70% (650000 million rials) during COVID-19. Given the growth of 79.5% in total income and 0.96% in operating income (compared to the growth of 49% in costs), the improvement of this ratio can be due to the credits received by the government during COVID-19 rather than the improvement of the FP of hospitals. The share of personnel costs, medicine, and medical supplies did not change from the total costs before and during COVID-19 (the share of medicines and consumables was 17%, and personnel costs were 68% of the total costs). Owing to the growth of salaries and incentives for medical staff and the lack of change in the composition of costs, the growth of medication costs and consumables did not change.

“Leverage ratios”: we found that all leverage ratios (ratio of total costs to total assets and debt to asset ratio) were positive during the pandemic. While the ratio of total costs to total assets and debt to asset ratio increased during COVID-19, and the current debt ratio decreased the current debt ratio decreased in 2019–20 before increasing during COVID-19. This slight increase during COVID-19 can be attributed to the growth of the share of government funding in the total revenues of the hospitals’ income (in the province) and the condition of COVID-19. The standard size of the current ratio per hospital is 1.75–2.75 [16], and a current ratio of < 1 means that the institution is unable to pay its short-term debt because its current assets are less than its current liabilities. Higher financial leverage is a sign of financial stress that could lead to bankruptcy and be associated with the lower investment [47, 48].

“Profitability ratios”: in this study, all profitability-related ratios were negative during COVID-19. The operating profit margin was negative and decreased in all hospitals during either or both pre-COVID or COVID periods. The amount of operating loss ratio means that for every one million rials of income from the sale of services by deducting operating costs, there was no profit, but there was a loss of 1.42 million rials. The decline in profit operating margin in Valiasr Hospital was the highest than other hospitals. Similar findings are reported by Yu Wang et al., which found that operating margins decreased in California hospitals overall during the first six quarters of the COVID-19 pandemic [49]. They also demonstrated that Despite operating losses, large increases in other operating revenue and non-operating revenue mitigated losses as government assistance programs and the stock market recovery took effect during the quarter [49]. Similarly, another study in the US showed, Covid-19 had a negative impact on hospital operating margin but not on the total margin. They also demonstrated a negative moderating role of staffed beds on hospital operating margin [50].

For any organization, a positive operating margin is essential for long-term survival. Few organizations can survive long when their total costs exceed their incomes. For hospitals, positive financial margins allow them to invest in new facilities, treatments, and technologies to better care for patients and create resources to recoup for unexpected costs or lack of income. Healthcare margins in hospitals were very low compared to other industries before COVID-19. A previous study showed that some US hospitals had negative margins before COVID-19. They lost their money in operating. In fact, the median hospital margin was a very modest 3.5% [51].

We also found net profit margin fluctuated in most hospitals and decreased in 2019–20 before increasing slightly during COVID-19, meaning that each one million rials of total income can have a loss equivalent to 0.04 million rials. Since in calculating net margin ratio, funds received from the government are included in total income and depend on credit distribution indicators, operating profit margin with operating profit formula (e.g., operating income- operating cost / operating income) can be a better basis for evaluating and comparing the FP of hospitals [52].

Also, ROA reduced in 2019–20 before rising in 2020–21. Although the two-year trend of ROA was descending pre-COVID-19 and changed from profit to loss, it increased during COVID-19 (The standard size of ROA is 0.025–0.15) [16]). A study in Indonesia demonstrated that ROA, current debt ratio, and the ratio of total debt to total assets increased during COVID-19 [16], which is consistent with our study.

Our findings also showed that liquidity ratios (cash ratio, current ratio, and quick ratio) experienced a decline during the pandemic. Poor capital liquidity affects the decision-making ability of public hospitals [53]. Public hospitals should ensure adequate capital liquidity to cope with the supplier’s accounts without any payment delay and have the ability to pay the medical staff [53]. COVID-19 impacted not only health systems but also other sections of countries worldwide. A study in Indonesia in 2020 showed while activity and leverage ratios were increased, liquidity and profitability ratios were decreased in state-owned companies during COVID-19. This study also showed that, although there were no significant differences between the liquidity and leverage ratios, a significant difference was found in the profitability and activity ratios between companies before and after COVID-19 [54]. It seems, more than ever, hospitals need government support and to reconsider their strategic financial plans for what is likely to be a very challenging environment, even with the reduction in COVID-19 cases [51].

### Limitations and strengths

Our study has limitations and strengths. One of this study’s strengths was to increase accuracy; we used monthly points to analyze the SP indicators such as BO, BTO, number of outpatients and inpatients, number of surgeries, deaths, ALOS, and inpatient bed days. Second, this was a multi-center study, involving all public hospitals of the province. Third, we evaluated both the financial and service performance of hospitals, and FP was evaluated through an optimal combination of financial indicators for sequential years. We also recognize some limitations of the study. Firstly, due to the lack of timely identification and registration of costs during the cost analysis, it was impossible to obtain information about the hospitals’ monthly costs. Secondly, due to the increase in equipment, consumables, etc., the costs were not adjusted to 2021 dollars. Thirdly, since the performance analysis of private hospitals was not taken into account, the performance analysis of public hospitals with private hospitals was not compared.

## Conclusion

Significant drops were observed in monthly volumes of inpatient bed day, BOR (%), BTO (patients), outpatients (N), inpatients (N), and surgeries (N). Similarly, a substantial decline was apparent in operating expense coverage from operating income and operating income of total revenue during either or both pre-COVID or COVID periods. Notably, in our study, although the total income per inpatient day rose during the pandemic, that increase was much lower compared with an increase in the total cost per inpatient day during the same period. Moreover, all profitability ratios were negative pre and during the COVID-19 outbreak. A significant reduction in the operating profit margin ratio and net profit margin during COVID-19 means that COVID-19 has harmed the FP of hospitals. However, an improvement in the net profit margin ratio positively impacts government financial support. All leverage ratios and activity ratios increased during either or both pre-COVID or COVID periods.

COVID-19 has decreased the FP and SP of hospitals due to limitations in providing services to patients since the beginning of COVID-19. Measures such as providing various financing resources and improving the financial resilience of hospitals are essential. Funds should be disbursed to offset hospitals’ losses due to reduced elective and outpatient revenue. Policymakers should come up with holistic policies to tackle the adverse impact of such crises in the future, support hospitals financially, and consider allocating additional funding to them during emergencies.

Our findings provide insights into the adverse effects of COVID-19 on hospitals. The results underscore that in addition to indirect costs (e.g. loss of job) that individuals may incur, hospitals incur a heavy burden during a global health crisis. Consequently, we emphasize the need for the government to support hospitals financially to compensate for financial losses during crises. Further, health policymakers and health managers should adopt preventive policies considering the adverse effects of a pandemic on hospitals in addition to the cost of the disease itself.

## Supporting information

S1 File STROBE statement—Checklist of items that should be included in reports of *cross-sectional studies*. (DOC)

## Abbreviations

SP: Service Performance
FP: Financial Performance
BTO: Bed Turnover
BOR: Bed Occupancy Ratio
ALOS: Average Length of Stay
HIS: Hospital Information System

## References

[1] Elias C, Nkengasong JN, Qadri F. Emerging Infectious diseases-learning from the past and looking to the future. The New England journal of medicine. 2021;384(13):1181–4. doi: 10.1056/NEJMp2034517 33793147

[2] Singh DR, Sunuwar DR, Shah SK, Karki K, Sah LK, Adhikari B, et al. Impact of COVID-19 on health services utilization in Province-2 of Nepal: a qualitative study among community members and stakeholders. BMC health services research. 2021;21(1):1–14. 33627115 10.1186/s12913-021-06176-y PMC7903406

[3] Mirkazehi Rigi Z, Dadpisheh S, Sheikhi F, Balouch V, Kalkali S. Challenges and Strategies to deal with COVID-19 from the perspective of physicians and nurses in southern of Sistan and Baluchestan, Iran. Journal Mil Med. 2020;22(6):599–606.

[4] Hofmeyer A, Taylor R. Strategies and resources for nurse leaders to use to lead with empathy and prudence so they understand and address sources of anxiety among nurses practising in the era of COVID‐19. Journal of clinical nursing. 2021;30(1–2):298–305. doi: 10.1111/jocn.15520 33006794 PMC7537231

[5] Barasa E, Kazungu J, Orangi S, Kabia E, Ogero M, Kasera K. Indirect health effects of the COVID-19 pandemic in Kenya: a mixed methods assessment. BMC Health Services Research. 2021;21(1):1–16. 34311716 10.1186/s12913-021-06726-4 PMC8311400

[6] Sun S, Xie Z, Yu K, Jiang B, Zheng S, Pan X. COVID-19 and healthcare system in China: challenges and progression for a sustainable future. Globalization and Health. 2021;17(1):1–8. 33478558 10.1186/s12992-021-00665-9 PMC7819629

[7] Mirzaei A, Rezakhani Moghaddam H, Habibi Soola A. Identifying the predictors of turnover intention based on psychosocial factors of nurses during the COVID‐19 outbreak. Nursing Open. 2021;8(6):3469–76. doi: 10.1002/nop2.896 33960721 PMC8242757

[8] Saki M, Ghanbari MK, Behzadifar M, Imani-Nasab MH, Behzadifar M, Azari S, et al. Focus: Preventive Medicine: The Impact of the Social Distancing Policy on COVID-19 Incidence Cases and Deaths in Iran from February 2020 to January 2021: Insights from an Interrupted Time Series Analysis. The Yale Journal of Biology and Medicine. 2021;94(1):13. 33795979 PMC7995950

[9] Behzadifar M, Aalipour A, Kehsvari M, Darvishi Teli B, Ghanbari MK, Gorji HA, et al. The effect of COVID-19 on public hospital revenues in Iran: An interrupted time-series analysis. Plos one. 2022;17(3):e0266343. doi: 10.1371/journal.pone.0266343 35358279 PMC8970352

[10] Abebe W, Worku A, Moges T, Tekle N, Amogne W, Haile T, et al. Trends of follow-up clinic visits and admissions three-months before and during COVID-19 pandemic at Tikur Anbessa specialized hospital, Addis Ababa, Ethiopia: an interrupted time series analysis. BMC Health Services Research. 2021;21(1):1–10. 34301264 10.1186/s12913-021-06730-8 PMC8301740

[11] Rennert-May E, Leal J, Thanh NX, Lang E, Dowling S, Manns B, et al. The impact of COVID-19 on hospital admissions and emergency department visits: A population-based study. PLoS One. 2021;16(6):e0252441. doi: 10.1371/journal.pone.0252441 34061888 PMC8168854

[12] Essien U R, Eneanya ND, Crews DC. Prioritizing equity in a time of scarcity: the COVID-19 Pandemic. Journal of general internal medicine. 2020;35(9):2760–2. doi: 10.1007/s11606-020-05976-y 32607930 PMC7325835

[13] Association AH. Hospitals and health systems face unprecedented financial pressures due to COVID-19. Available from: https://wwwahaorg/guidesreports/2020-05-05-hospitals-and-health-systems-face-unprecedented-financial-pressures-due. 2020.

[14] Khalilpourazari S, Hashemi Doulabi H. Robust modelling and prediction of the COVID-19 pandemic in Canada. International Journal of Production Research. 2021:1–17.

[15] Jamaati H, Dastan F, Varahram M, Hashemian SM, Rayeini SN, Farzanegan B, et al. COVID-19 in Iran: a model for crisis management and current experience. Iranian Journal of Pharmaceutical Research: IJPR. 2020;19(2):1. doi: 10.22037/ijpr.2020.113365.14255 33224206 PMC7667532

[16] Yuniarti R, Paryanti D, Tejaningsih A. Analysis of Financial Performance and Services Performance Before and During the Covid-19 Pandemic (Case Study At Bayu Asih Hospital Purwakarta). Turkish J Physiother Rehabil. 2020;32(3):6103–12.

[17] Khullar D, Bond AM, Schpero WL. COVID-19 and the financial health of US hospitals. Jama. 2020;323(21):2127–8. doi: 10.1001/jama.2020.6269 32364565

[18] dan Houston B. Dasar-dasar Manajemen Keuangan Buku 1 (edisi II). Jakarta: Salemba Empat. 2010.

[19] Ichsan R, Suparmin S, Yusuf M, Ismal R, Sitompul S. Determinant of Sharia Bank’s Financial Performance during the Covid-19 Pandemic. Budapest International Research and Critics Institute-Journal (BIRCI-Journal). 2021:298–309.

[20] Sudana IM. Manajemen Keuangan Perusahaan (Edisi 2). Jakarta: Erlangga. 2015.

[21] Byun H, Kang D, Go S-I, Kim HI, Hahm JR, Kim RB. The impact of the COVID-19 pandemic on outpatients of internal medicine and pediatrics: A descriptive study. Medicine. 2022;101(8). 35212289 10.1097/MD.0000000000028884 PMC8878857

[22] Kaufman HW, Chen Z, Niles J, Fesko Y. Changes in the number of US patients with newly identified cancer before and during the coronavirus disease 2019 (COVID-19) pandemic. JAMA network open. 2020;3(8):e2017267–e. doi: 10.1001/jamanetworkopen.2020.17267 32749465 PMC7403918

[23] Ateev Mehrotra MC, David Linetsky, Hilary Hatch DC. The Impact of the COVID-19 Pandemic on Outpatient Visits: A Rebound Emerges. Available from: https://wwwcommonwealthfundorg/publications/2020/apr/impact-covid-19-outpatient-visits. 2021.

[24] Wang W, Zheng Y, Jiang L. Impact of the COVID-19 epidemic on outpatient visits of common respiratory diseases. 2020.

[25] Katz SE, Spencer H, Zhang M, Banerjee R. Impact of the COVID-19 pandemic on infectious diagnoses and antibiotic use in pediatric ambulatory practices. Journal of the Pediatric Infectious Diseases Society. 2021;10(1):62–4. doi: 10.1093/jpids/piaa124 33064837 PMC7797736

[26] King LM, Lovegrove MC, Shehab N, Tsay S, Budnitz DS, Geller AI, et al. Trends in US outpatient antibiotic prescriptions during the coronavirus disease 2019 pandemic. Clinical Infectious Diseases. 2021;73(3):e652–e60. doi: 10.1093/cid/ciaa1896 33373435 PMC7799289

[27] Birkmeyer JD, Barnato A, Birkmeyer N, Bessler R, Skinner J. The impact of the COVID-19 pandemic on hospital admissions in the United States: study examines trends in US hospital admissions during the COVID-19 pandemic. Health Affairs. 2020;39(11):2010–7. 32970495 10.1377/hlthaff.2020.00980 PMC7769002

[28] Wambua S, Malla L, Mbevi G, Nwosu A-P, Tuti T, Paton C, et al. The indirect impact of COVID-19 pandemic on inpatient admissions in 204 Kenyan hospitals: An interrupted time series analysis. PLOS Global Public Health. 2021;1(11):e0000029. doi: 10.1371/journal.pgph.0000029 36962093 PMC10021711

[29] Heydarian M, Behzadifar M, Chalitsios CV, Keshvari M, Omidifar R, Ghanbari MK, et al. Effect of COVID-19 on the number of CT-scans and MRI services of public hospitals in Iran: an interrupted time series analysis. Ethiopian Journal of Health Sciences. 2021;31(6). doi: 10.4314/ejhs.v31i6.5 35392347 PMC8968382

[30] Hung M, Mennell B, Christensen A, Mohajeri A, Azabache H, Moffat R. Trends in COVID-19 Inpatient Cases and Hospital Capacities during the Emergence of the Omicron Variant in the United States. COVID. 2022;2(9):1207–13.

[31] Khan JR, Awan N, Islam MM, Muurlink O. Healthcare capacity, health expenditure, and civil society as predictors of COVID-19 case fatalities: a global analysis. Frontiers in public health. 2020;8:347. doi: 10.3389/fpubh.2020.00347 32719765 PMC7349997

[32] Cavallo JJ, Donoho DA, Forman HP, editors. Hospital capacity and operations in the coronavirus disease 2019 (COVID-19) pandemic—planning for the Nth patient. JAMA health forum; 2020: American Medical Association. 10.1001/jamahealthforum.2020.0345 36218595

[33] Azari S, Omidi N, Arabloo J, Pourhosseini H, Rezapour A. Resource utilization and cost of hospitalized patients with COVID-19 in Iran: rationale and design of a protocol. Frontiers in Emergency Medicine. 2020;4(2s):e55–e.

[34] Kazempour-Dizaji M, Sheikhan F, Varahram M, Rouzbahani R, Vand MY, Khosravi B, et al. Changes in a hospital’s costs and revenues before and after COVID-19: A case study of an Iranian hospital. Health Scope. 2021;10(3).

[35] Hassan-Nezhad B, Moosavi-Nezhad SM, Enayat H. Assessing the financial performance of hospitals in the Covid-19 crisis: a case study of a hospital in Tehran. EBNESINA. 2021;23(3):72–8.

[36] Sajjadi Khasraghi J, Salesi M, Meskarpour Amiri M, Mohammadian M, Khosmanzar J, Abdi M. The impact of the COVID-19 pandemic on the financial and performance indicators of hospitals: A case study in Tehran, Iran. Iranian Journal of Health Insurance. 2022;5(3):238–46.

[37] Lino DOdC, Barreto R, Souza FDd, Lima CJMd, Junior GBdS. Impact of lockdown on bed occupancy rate in a referral hospital during the COVID-19 pandemic in northeast Brazil. Brazilian Journal of Infectious Diseases. 2020;24:466–9. 10.1016/j.bjid.2020.08.002 PMC7457936 32888904

[38] Castagna F, Xue X, Saeed O, Kataria R, Puius YA, Patel SR, et al. Hospital bed occupancy rate is an independent risk factor for COVID-19 inpatient mortality: a pandemic epicentre cohort study. BMJ open. 2022;12(2):e058171. doi: 10.1136/bmjopen-2021-058171 35168984 PMC8852235

[39] Nurettin Oner M. Organizational and environmental factors associated with hospital financial performance: A systematic review. Journal of Health Care Finance. 2016;43(2).

[40] Troeger C. Just How Do Deaths Due to COVID-19 Stack Up? Available from: https://wwwthinkglobalhealthorg/article/just-how-do-deaths-due-covid-19-stack. 2021.

[41] Lucero A, Sokol K, Hyun J, Pan L, Labha J, Donn E, et al. Worsening of emergency department length of stay during the COVID‐19 pandemic. Journal of the American College of Emergency Physicians Open. 2021;2(3):e12489. doi: 10.1002/emp2.12489 34189522 PMC8219281

[42] Blumenthal D, Fowler EJ, Abrams M, Collins SR. Covid-19—implications for the health care system. Mass Medical Soc; 2020. p. 1483–8. doi: 10.1056/NEJMsb2021088 32706956

[43] Project TC-T. The Data. Available from: https://protect-publichhsgov/pages/hospital-capacity Accessed 3 April 2023. 2021.

[44] Barten DG, Kusters RW, Peters NA. A swift and dynamic strategy to expand emergency department capacity for COVID-19. Disaster Medicine and Public Health Preparedness. 2022;16(3):1190–3. doi: 10.1017/dmp.2020.430 33143801 PMC7884671

[45] Lucero AD, Lee A, Hyun J, Lee C, Kahwaji C, Miller G, et al. Underutilization of the emergency department during the COVID-19 pandemic. Western Journal of Emergency Medicine. 2020;21(6):15. doi: 10.5811/westjem.2020.8.48632 33052821 PMC7673895

[46] Bai G, Zare H. Hospital cost structure and the implications on cost management during COVID-19. Journal of general internal medicine. 2020;35(9):2807–9. doi: 10.1007/s11606-020-05996-8 32607935 PMC7326305

[47] Lombardi M O’Connor SJ, Carroll N, Szychowski JM, Nancy Borkowski D. The Relationship of Debt Ratio and Financial Performance for Large Not-for-Profit Health Systems. Journal of Health Care Finance. 2021.

[48] OECD. Insolvency and debt overhang following the COVID-19 outbreak: assessment of risks and policy responses. Available from: https://wwwoecdorg/coronavirus/policy-responses/insolvency-and-debt-overhang-following-the-covid-19-outbreak-assessment-of-risks-and-policy-responses-7806f078/ Accessed 3 April 2023. 2020.

[49] Wang Y, Witman AE, Cho DD, Watson ED, editors. Financial Outcomes Associated With the COVID-19 Pandemic in California Hospitals. JAMA health forum; 2022: American Medical Association. 10.1001/jamahealthforum.2022.3056 PMC9508652 36218945

[50] He M, Jessri M, Zhang H. The impact of COVID-19 on hospitals’ financial performance: Evidence from California hospitals. International Journal of Healthcare Management. 2022:1–8.

[51] Hall K. The effect of COVID-19 on hospital financial health. Available from: https://wwwkaufmanhallcom/sites/default/files/documents/2020-07/Effect-COVID19-Financial-Health_KaufmanHallpdf. 2020.

[52] Burkhardt JH, Wheeler JR. Examining financial performance indicators for acute care hospitals. Journal of health care finance. 2013;39(3):1–13. 23614262

[53] Hu X, Jin W, Yang A, Hu Z. Management of capital liquidity in public hospitals under the epidemic situation of COVID-19. Frontiers in public health. 2022;10. doi: 10.3389/fpubh.2022.977221 36339180 PMC9631788

[54] Devi S, Warasniasih NMS, Masdiantini PR, Musmini LS. The impact of COVID-19 pandemic on the financial performance of firms on the Indonesia stock exchange. Journal of Economics, Business, & Accountancy Ventura. 2020;23(2):226–42.

